# Fractional Exhaled Nitric Oxide (FeNO) in Biomass Smoke-Associated Chronic Obstructive Pulmonary Disease

**DOI:** 10.3390/medsci12040052

**Published:** 2024-10-04

**Authors:** Juan Silva-Gallardo, Raúl H. Sansores, Alejandra Ramírez-Venegas, Robinson E. Robles Hernández, Gustavo I. Centeno-Saenz, Rafael J. Hernández-Zenteno

**Affiliations:** 1 Hospital General Regional 25, Mexican Social Security Institute, Mexico City 09100, Mexico; mely0306@yahoo.com.mx; 2 Respiratory Department, Médica Sur Hospital, Mexico City 14050, Mexico; raulsansores@yahoo.com.mx; 3 Tobacco and COPD Research Department, Ismael Cosío Villegas National Institute of Respiratory Diseases, Mexico City 14080, Mexico; aleraves@gmail.com (A.R.-V.); robinsonrobher@gmail.com (R.E.R.H.); igcenteno89@gmail.com (G.I.C.-S.); 4 COPD and Bronchiectasis Clinic, Ismael Cosío Villegas National Institute of Respiratory Diseases, Mexico City 14080, Mexico

**Keywords:** biomass COPD, FeNO, inflammation

## Abstract

Chronic Obstructive Pulmonary Disease (COPD) is a disease characterized by local and systemic inflammation independently of the risk factor; during the exacerbations, such inflammation is accentuated and amplified. A practical inflammatory marker and one with an applicable predictive value in the follow-up has been sought. FeNO has shown an excellent performance in that respect within the context of asthma and has also been studied in tobacco-smoke COPD (COPD-TS). In Biomass-smoke COPD (COPD-BS), this, to our knowledge, has not been evaluated. Objective: To measure FeNO levels in patients with COPD-BS and to compare these with those of patients with stable COPD-TS and in healthy controls. Methods: Transversal, observational, descriptive, comparative, and analytical study. A total of 57 patients, including 23 with COPD-BS, 17 with COPD-TS, and 17 healthy control subjects. The measurement of FeNO was carried out on all of these by means of the on-line chemiluminescence technique; the values were expressed in parts per billion (ppb) for their analysis. Results: It was observed that the FeNO values were similar between COPD-BS and COPD-TS and were significantly different between the healthy and stable COPD (both groups). No correlation was found between pulmonary function and symptoms with FeNO in any of the groups. Conclusions: The level of FeNO in stable COPD is found to be increased in a similar manner in COPD-BS and COPD-TS, with a significant difference on comparing it with that of the healthy subjects.

## 1. Introduction

Chronic obstructive pulmonary disease (COPD) is an inflammatory disease characterized by the partially reversible obstruction of air flow. It is generally accompanied by mucous hypersecretion and oxidative stress in the airways and in the lungs [1]. The principal cause of COPD at the worldwide level is tobacco smoking (TS). However, in developing countries, another risk factor comprises chronic exposure to biomass (wood) smoke (BS), which presents, above all, in women who employ this energy source to cook and to heat their homes [2]. Although both types of COPD share physiological changes, important functional and clinical differences have been described [3,4]. Fractional exhaled nitric oxide (FeNO) is one of the inflammatory biomarkers that is synthesized in the airways, and it plays a key role in physiological regulation, acting as neurotransmitter, vasodilator, and in immunoregulation [5]. The increase of FeNO has been widely described and studied in inflammatory disease of the airways; mainly in asthma, cystic fibrosis, and pulmonary hypertension [6]. Measuring FeNO in the exhaled air in inflammatory diseases of the airways has become an attractive auxiliary tool in the detection and follow-up of these diseases because it is a simple, non-invasive method that has demonstrated clinical utility and that has been standardized and recommended by the American Thoracic Society (ATS) for the study and follow-up of patients with asthma both in children and adults [7]. However, its usefulness in COPD continues to be controversial. Although there are works that have investigated diverse profiles of FeNO, the results have been disappointing and inconsistent as it relates to COPD, giving rise to confusion [8]. There are, to our knowledge, no studies that have evaluated the behavior of this biomarker in COPD-BS. One might think that, because the phenotype of COPD-BS is more attributable to the airway, and not to emphysema, these levels could be found more elevated in these patients on comparison with those of COPD-TS.

To explore this association, we carried out this study in a sample of patients previously diagnosed with COPD-BS in a cohort of patients at the Mexican National Institute of Respiratory Diseases (INER) seen at their outpatient service. We compared the results with those of patients with COPD-TS, with both groups currently in stable condition. The study also included a control group of healthy adults aged over 40 years to contrast the results.

## 2. Materials and Methods

### 2.1. Study Population

The study was carried out in Mexico City in the National Institute of Respiratory Diseases (INER) at the COPD Clinic Patient Consultation Service. We recruited three groups of patients (COPD-BS, COPD-TS, and healthy subjects who had received a FeNO measurement). The healthy subjects were recruited from the volunteer hospital personnel, or from among the relatives of the patients with sociodemographic characteristics similar to those of the patients with COPD.

A brief questionnaire was applied to all control subjects to ensure that they were healthy, did not currently or previously smoke tobacco, did not experience any other addiction type, and did not have any type of respiratory disease, atopies, or chronic rhinosinusitis. 

All of these individuals were invited to participate by means of a telephone call. On a capture sheet, we obtained their general data, clinical state, the data from their last spirometry, the etiology of the COPD (tobacco smoking or wood smoke), the state of the smoker, with their index of smoking and/or exposure to wood smoke, the severity of the stable disease according to standard “GOLD” criteria, the use of inhaled or systemic steroids, the presence of comorbid processes, and the degree of dyspnea based on the scale of the modified Medical Research Council (mMRC). All of the patients signed the informed consent document to participate in the study, which was reviewed and approved by both the local ethics review boards and the Institutional Review Board (Protocol C08–05) in accordance with the principles of the Declaration of Helsinki. The tests were performed during the morning hospital shift by the principal researcher (SGJ).

The values of FeNO were captured in a database and were expressed in parts per billion (ppb) for their analysis.

### 2.2. Measurement Technique of Nitric Oxide

We employed the on-line chemiluminescence measurement technique after a prior review of the Users’ Manual of the brand of the equipment utilized (NO Analyzer-Sievers™, version 3.2 software) (Boulder, CO, USA). We conducted a daily calibration of the equipment prior to carrying out the test according to the manufacturer’s recommendations.

We followed the recommendations for the ATS and European Respiratory Society (ERS) tests in adults [9]. We utilized the Windows XP Professional x64 Edition system. This method employs a constant flow velocity of 50 ± 5 mL/s. The measurements were always performed prior to 10 A.M., by asking the patient to exhale the air from their total lung capacity, and the patient received an indication at the moment of carrying out the test to observe a chromatic bar indicator displaying flow and pressure on the computer screen. The objective of this was to maintain the expiratory flow for the longest time possible (at least 6 s) and to be able to reach a plateau of 3 s. In addition, to avoid leaks, the patients were asked to maintain a hermetic seal between their lips and the specific mouthpiece filter; with the latter, a pressure level in the mouth is generated of between 5 and 20 cm H_2_O, which allows for the closure of the *veli palatini*, thus avoiding contamination with nasal gas.

**Figure 1:**
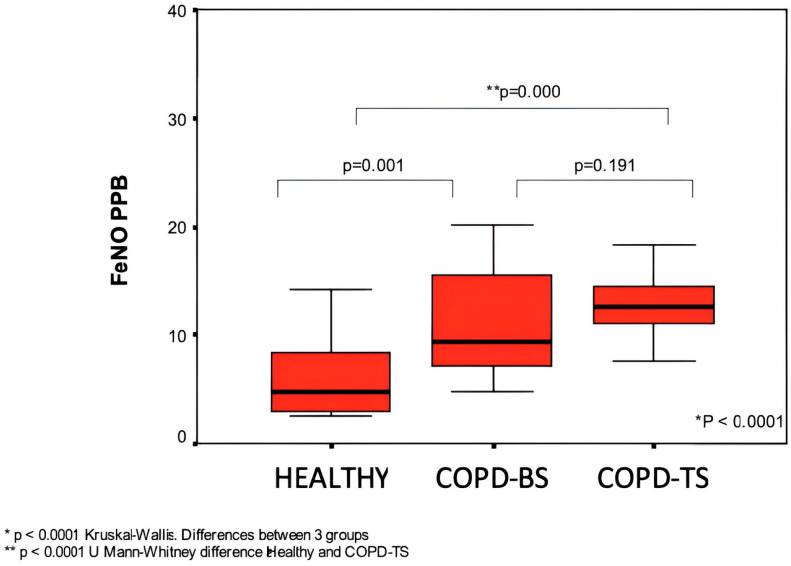
Comparison of FeNO in stable patients versus healthy patients.

All study participants had at least three acceptable measurements at intervals of at least 3 min between each measurement. The values measured were considered acceptable if the variability between these measurements was less than 5 percent. The average value of the two highest measurements was considered the final FeNO (Figure 1).

### 2.3. Statistical Analyses

Parametric tests were employed to describe the general population of the patients using means and standard deviations (SD). Comparison of the demographic variables among the three groups of study participants was carried out by the analysis of repeated samples (analysis of variance, ANOVA). Because the measurement of FeNO did not maintain a normal distribution, we utilized a non-parametric analysis for repeated samples (Kruskal–Wallis). To determine where the differences were found, we performed non-paired analyses utilizing the un-paired student T test or the Mann–Whitney U test according to the distribution of the data. To ascertain whether some correlation existed between FeNO, the degree of obstruction, and the measurement of dyspnea, we utilized the Pearson R correlation test.

## 3. Results

In Table 1, we find the demographic data of the patients included in the study. Significant differences were observed in the forced expiration value (FEV)1 (*p* < 0.03) between the two groups of patients with COPD. Around 50% of the patients were classified as GOLD IV. The treatments with bronchodilators and steroids were similar for both groups. In the two groups, around 60% were utilizing inhaled steroids. The results of the FeNO measurements of the two COPD groups and the healthy control group are presented in Figure 1, which illustrates that there were statistically significant differences among the healthy subjects and the two COPD groups (*p* = 0.001), but not between the COPD groups (*p* < 0.2).

There was no correlation between pulmonary function and dyspnea with the levels of FeNO for any of the groups. We also conducted a multiple linear regression analysis using log-transformed FeNO as the outcome variable and sex, age, height, FEV1%, steroid use, and exposure groups (BS, TS, HC) as predictors. The model explained 25% of the variance in log-FeNO values (R^^2^^ = 0.2502, *p* = 0.0910).

The difference in FeNO levels between smokers and non-smokers was statistically significant, with a mean difference of −4.6 ppb and a standard deviation of 1.9 (*p* = 0.021). Additionally, when comparing current smokers, non-smokers, and former smokers, the *p*-value was 0.0022, indicating a significant difference between these groups as well.

## 4. Discussion

This is, to our knowledge, the first study that measures the values of the nitric-oxide levels (NO) in COPD-BS and that compares these with those of COPD-TS. This study demonstrates that the levels of FeNO in patients with COPD-BS are similar to those in patients with COPD-TS found in stable condition, suggesting that they share the same physiopathological processes of inflammation and oxidative stress.

An overlap was observed of the levels of FeNO between the healthy control-group subjects and the two groups of stable COPD, as has been reported in a meta-analysis that evaluated FeNO in patients with stable COPD who were also compared with healthy controls [5]. In this systemic review and meta-analysis, the authors describe only from 1998–2017; in 18 studies, the measurement was conducted with the chemiluminescence technique, while in 13 studies, FeNO levels were higher than those in the healthy control group, while in the remainder of the studies, there was no difference.

We did not find an association between pulmonary function and the FeNO level, as reported by Beg and Fan et al. [8,10], and only observed a correlation between COPD in relation to Forced Expiration Volume at 1 s (FEV1)/Forced Vital Capacity (FVC). We also did not find a correlation between FeNO levels, and the disease severity of COPD based on the degree of obstruction as found by Fan et al. [10].

We did not include patients with exacerbated COPD. Few studies have evaluated FeNO in stable condition, where attention is drawn to that in a stable condition, the FeNO level was even higher than in exacerbated patients (41 vs. 13 ppb) [5]. Another interesting observation lies in that ex-smokers express a lesser concentration of FeNO (2 ppb less) than active smokers, because tobacco smoke inhibits the inducible isoform of nitric oxide synthase; in our population, the majority of the patients were no longer exposed to tobacco or to the biomass [5].

The patients with COPD-BS had a FeNO level similar to that of patients with COPD-TS; these findings suggest that, despite that the COPD-BS phenotype is predominantly through the airway, it could not be a biomarker to distinguish the most accentuated damage of the airway in this phenotype.

Although the standardized FeNO measurement technique by ATS/ERS was that of on-line chemiluminescence in 2005, in recent years novel portable devices with different technologies have been validated by the U.S. Food and Drug Administration (FDA), due to their high correlation with the standardized technique considered the “GOLD” standard, which is chemiluminescence, reporting an excellent correlation in persons with asthma and in healthy subjects (r = 0.94 and 0.96, respectively) [11], in that the equipment for chemiluminescence is desk-oriented and utilizes a computer, rendering it of easy, at-hand access for this measurement. This draws our attention to that our values measured with FeNO are in general lower than the measurements in the majority of studies conducted on this subject matter, which we attribute probably to three possible reasons. The majority of patients with COPD-BS were women who were on average short in height, which is a factor that modifies the level of FeNO [7]. When the value of FeNO has been compared between men and women, it is lower in women (16.8 vs. 14.3 ppb, a difference of 2.5 ppb) [12]; it could be that the lungs of the shortest women exhibit a lower surface expression of NO.

Approximately one half of the patients in both groups used inhaled steroids in their treatment, the use of corticosteroids was necessary to manage their advanced COPD symptoms and reduce inflammation, particularly in those with frequent exacerbations. Most of the subjects initiate medication according to GOLD report recommendations (LABA/ICS) before to “Triple Therapy Era”. The study of Fan et al. [10], which evaluated hospitalized exacerbated patients with asthma, demonstrated that after a course of systemic steroids, there is a slight but significant diminution of FeNO. However, the employment of inhaled steroids has not, to our knowledge, been evaluated in patients with COPD. Finally, the measurements were performed at an average altitude of 2250 m above sea level. It is known that altitude entertains an inverse correlation with FeNO, as reported by Ren et al. [12], who demonstrated, in healthy adult subjects in some studies in Tibet, a mean of 15.4 ppb.

One of the limitations of this study was not having performed spirometries in the healthy subjects, in whom we had objectively discarded the presence of obstruction or functional restriction, nor was a blood test for eosinophils or chest X-ray performed, but lung disease and exposure to tobacco or wood smoke were ruled out through a standardized questionnaire. Another limitation of our study is that we did not conduct measurements of eosinophils in blood or in sputum, and of immunoglobulin E, which could have aided in investigating possible atopies in some patients.

Another limitation of our study was that the measurement of FeNO was unique for each study participant; however, it has been demonstrated that the individual variability of this test did not exhibit significant differences (3.9 ppb) when measured at different moments in the short term [13]. Additionally, patients were treated based on the 2005 GOLD guidelines available at the time, where the use of inhaled corticosteroids (ICS) was only recommended in combination with LABA/ICS.

On the other hand, the sample size of the patients studied is small; thus, it could not be representative of the findings reported. In addition, the heterogeneity of the groups since the number of healthy controls and those exposed to tobacco was lower.

## 5. Conclusions

We conclude that the levels of FeNO in patients with stable COPD-BS are similar to the values reported in COPD-TS. More studies should be conducted in order to elucidate the role of FeNO in COPD-BS.

## Figures and Tables

**Table 1:** Comparison between the two stable groups: COPD-BS vs. COPD-TS vs. the healthy controls group.

Variables	COPD-BS *n* = 23	COPD-TS *n* = 17	HEALTHY *n* = 17	*p* Value
Demographic data				
M/F Gender (% M)	1/22 (4.3)	13/4 (76.5)	3/14 (17.6)	<0.0001
Age (years)	74.0 ± 7.18	73.5 ± 8.2	52.4 ± 12.3	<0.0001
Risk factors				
Smoking *n* (%)				
Currently	--	2/17 (11.8)	--	<0.0001
Ex-smoker	--	15/17 (88.2)	2/17 (11.8)	
Never-smoker	23/23 (100.0)	--	15/17 (88.2)	
Smoking index (packs per year)	--	44.05 ± 24.0	--	--
Wood exposure index (years)	291.68 ± 139.39	--	--	--
Pulmonary function data				
GOLD *n* (%)				
0–II	9/23 (21.7)	6/17 (11.7)	--	0.542
III–IV	14/23 (4.3)	11/17 (11.7)	--	
Dyspnea mMRC *n*%				0.017
0–1	6/22 (27.3)	8/17 (47.1)		
2–4	16/22 (72.7)	9/17 (52.9)		
FEV1, L	1.14 ± 0.34	1.35 ± 0.51	--	0.16
FEV1% predicted	70.80 ± 20.64	54.81 ± 23.08	--	0.035
FVC % predicted	85.00 ± 15.94	78.5 ± 25.51	--	0.356
FEV1/FVC %	64.90 ± 12.77	52.00 ± 13.08	--	0.005
Steroid use Yes/No				
(%Yes)	15/6 (65.2)	10/5 (59.0)	--	0.521
FeNO (ppb) median (range IC)	9.40 (4.8–28.2)	12.6 (7.5–34.9)	4.8 (2.5–14.1)	<0.0001

Proportions *n*%, mean ± standard deviation (SD) or median with interquartile (IQ) ranges according to the variable.

## Data Availability

The datasets used and/or analyzed during the current study are available from the corresponding author upon reasonable request.
